# Detection of Loss of Heterozygosity in cfDNA of Advanced *EGFR*- or *KRAS*-Mutated Non-Small-Cell Lung Cancer Patients

**DOI:** 10.3390/ijms21010066

**Published:** 2019-12-20

**Authors:** Elisa Boldrin, Giorgia Nardo, Elisabetta Zulato, Laura Bonanno, Valentina Polo, Stefano Frega, Alberto Pavan, Stefano Indraccolo, Daniela Saggioro

**Affiliations:** 1 Immunology and Molecular Oncology Unit, Istituto Oncologico Veneto IOV-IRCCS, Via Gattamelata 64, 35128 Padova, Italy; elisa.boldrin@iov.veneto.it (E.B.); giorgia.nardo@iov.veneto.it (G.N.); elisabetta.zulato@iov.veneto.it (E.Z.); daniela.saggioro@iov.veneto.it (D.S.); 2 Medical Oncology 2, Istituto Oncologico Veneto IOV-IRCCS, Via Gattamelata 64, 35128 Padova, Italy; laura.bonanno@iov.veneto.it (L.B.); stefano.frega@iov.veneto.it (S.F.); alberto.pavan@iov.veneto.it (A.P.); 3 Medical Oncology, Azienda ULSS 2, 31100 Treviso, Italy; valentina.polo@aulss2.veneto.it

**Keywords:** non-small-cell lung cancer (NSCLC), liquid biopsy, loss of heterozygosity (LOH), cell-free DNA (cfDNA), epidermal growth factor receptor (*EGFR*), Kirsten rat sarcoma homologous (*KRAS)*

## Abstract

Liquid biopsy is currently approved for management of epidermal growth factor receptor (*EGFR*)-mutated non-small-cell lung cancer (NSCLC) patients. However, one unanswered question is whether the rate of cell-free DNA (cfDNA)-negative samples is due to technical limitations rather than to tumor genetic characteristics. Using four microsatellite markers that map specific chromosomal loci often lost in lung cancer, we conducted a pilot study to investigate whether other alterations, such as loss of heterozygosity (LOH), could be detected in *EGFR*-negative cfDNA. We analyzed *EGFR*-mutated NSCLC patients (*n* = 24) who were positive or negative for *EGFR* mutations in cfDNA and compared the results with a second cohort of 24 patients bearing *KRAS*-mutated cancer, which served as a representative control population not exposed to targeted therapy. The results showed that in *EGFR*-negative post-tyrosine-kinase-inhibitor (TKI) cfDNAs, LOH frequency was significantly higher than in both pre- and post-TKI *EGFR*-positive cfDNAs. By contrast, no association between *KRAS* status in cfDNA and number of LOH events was found. In conclusion, our study indicates the feasibility of detecting LOH events in cfDNA from advanced NSCLC and suggests LOH analysis as a new candidate molecular assay to integrate mutation-specific assays.

## 1. Introduction

Non-small-cell lung cancer (NSCLC) is the leading cause of cancer-related death worldwide and has a five-year survival rate of less than 10% in patients with advanced disease [1]. The last decade has seen considerable changes in the field of systemic and molecular characterization of NSCLC. The discovery of oncogenic driver mutations and the concept of oncogene addiction modified the therapeutic approach for patients with advanced NSCLC [2]. Specifically, the identification of activating mutations in the epidermal growth factor receptor (*EGFR*) gene (present in approximately 15% of Caucasian patients) introduced the era of targeted therapy in advanced NSCLC, shifting treatment from platinum-based chemotherapy to tyrosine kinase inhibitors (TKIs) to manage first-line advanced disease [3]. Other potential therapeutic targets, including rearrangements involving *ALK*, *ROS1*, or *RET*, and mutations of *BRAF*, *ERBB2*, and *MET* have been identified in lung adenocarcinoma, although their incidence is low (range of 1–5%) [4]. In contrast, other genes such as *TP53* and *KRAS,* the mutations of which are among the most prevalent alterations in NSCLC (detected in 40–50% and 25–35% of patients, respectively), currently remain orphans of approved targeted therapies [5].

As biopsy specimens can be inadequate for routine genetic profiling in up to 30% of cases [6], circulating cell-free DNA (cfDNA) analysis, also referred to as liquid biopsy, has emerged as a new tool for detecting clinically relevant genetic alterations in lung cancer patients. Indeed, in advanced NSCLC, liquid biopsy is now used as a diagnostic assay to investigate *EGFR*-sensitizing mutations at baseline, when tissue is not adequate, or to detect the acquired resistance EGFR T790M mutation at disease progression [7]. Moreover, the ability to identify and quantify tumor-associated alterations in cfDNA by next-generation sequencing (NGS) technologies is opening up new opportunities to better characterize tumor heterogeneity and evolution [8].

According to a recent meta-analysis [9], the average sensitivity of *EGFR* liquid biopsy when using routine CE IVD-approved qPCR methods is 65–70%, although real-world studies have shown that the performance of cfDNA testing varies significantly between different laboratories [10]. We recently conducted a monocentric study, termed EMULATING (*EGFR* MUtation status from pLAsma: circulaTING tumor DNA analysis in NSCLC patients), which investigated the ability to detect in plasma of advanced NSCLC patients *EGFR* mutations previously identified in matched tumor tissue biopsy. We found that when using targeted PCR-based methods, detection sensitivity was 56%, which is substantially in line with literature data.

One unanswered question in the field is whether samples lacking detectable *EGFR* mutations in cfDNA (including both sensitizing and resistance mutations) are invariably due to the limited sensitivity of standard methods. This possible explanation is supported by the observation that the sensitivity of liquid biopsy analysis when using very high sensitivity methods, including BEAMing or droplet digital PCR (ddPCR), can be >80% [9]. Therefore, one could speculate that, as technology improves steadily, the gap between the sensitivity of *EGFR* tests in tumor tissue versus cfDNA will be narrower in the future. On the other hand, an alternative hypothesis, not mutually exclusive with the former, is that the lack of an *EGFR* mutation in plasma might be related to tumor heterogeneity and that treatment with TKI favors in some patients bearing an *EGFR*-mutated tumor a progressive emergence of a subclone(s) lacking *EGFR* mutations. This tumor cell population, which is likely negligible at diagnosis, fitting the general view that *EGFR* mutations are in the genetic trunk of the tumor [11], could be selected during therapy with EGFR TKI. In this scenario, one would expect to find other genetic fingerprints of the tumor in cfDNA despite not being able to detect *EGFR* mutations in the plasma sample.

In this study, we investigated this hypothesis by interrogating loss of heterozygosity (LOH) in cfDNA of NSCLC patients using four microsatellite (MS) markers that map specific chromosomal loci often lost in a broad range of tumors [12,13,14,15], including lung cancer [16,17]. LOH involves loss of wild-type allele unmasking the presence of inactivating mutations in the remaining allele; thus, its frequency is generally similar to the frequency of the mutation in a hot spot region. On the contrary, when we are faced with multiple low-frequency mutations in a given locus, LOH occurrence is usually higher than any individual mutation, thus maximizing the rate of alteration detection [18].

LOH was analyzed in a cohort of *EGFR*-mutated NSCLC patients who were positive or negative for *EGFR* mutations in their cfDNA. As a control, patients carrying *KRAS* mutations, commonly present in the vast majority of NSCLC cells, were also analyzed.

## 2. Results

### 2.1. Patients

Forty-eight patients with advanced NSCLC from EMULATING and MAGIC-1 (Monitoring Advanced NSCLC through plasma Genotyping during Immunotherapy: Clinical feasibility and application) trials were included in the study. Twenty-four were positive for *EGFR* mutations (*EGFR-*mutated) and 24 for *KRAS* mutations (*KRAS-*mutated) in tumor specimens. Clinical and pathological features of the 48 NSCLC selected patients are reported in Table 1. Briefly, the median age of *EGFR*-mutated patients was 70 years (range of 43–87), with a slight prevalence of males (*n* = 13; 54%). Smoking history was negative in about half the sample (*n* = 13; 54.1%), whereas 11 patients (45.8%) were former smokers. At the time of diagnosis, patients presented in an optimal or good Eastern Cooperative Oncology Group (ECOG) performance status (PS), with 6 (25%) and 18 (75%) having ECOG 0 and 1, respectively. Patients were included in the study when diagnosed as advanced disease (IIIb–IV, according to VII TNM (Table 1A)). Among *KRAS*-mutated patients, the median age was 69.5 years (range of 48–85); the majority of patients were female (*n* = 13; 54%). Almost all patients (*n* = 20; 83.3%) had been exposed to smoke, with 37.5% (*n* = 9) current smokers and 45.8% (*n* = 11) former smokers at time of diagnosis. Similar to the *EGFR*-mutated cohort, nearly all patients presented optimal or good PS (ECOG 0, *n* = 8, 33%; ECOG 1, *n* = 15, 63%). All patients had stage III–IV at the time of diagnosis (Table 1B).

**Figure 1:**
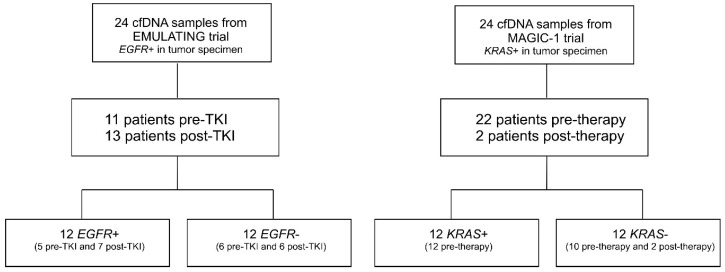
Flowchart of the study.

As described in Figure 1, among cfDNAs from *EGFR*-mutated patients, 11 were collected before TKI therapy (pre-TKI), whereas 13 were obtained during treatment (hereinafter referred to as post-TKI). Twelve cfDNAs carried the same *EGFR* mutation found in tumor tissue (*EGFR+*), whereas the remaining 12 samples were *EGFR* mutation negative (*EGFR*−). Regarding the 24 cfDNAs from *KRAS*-mutated patients, all samples were collected before the start of systemic treatment. As reported in Figure 1, 12 cfDNAs were *KRAS*+ and 12 were *KRAS*−.

### 2.2. Analysis of LOH in EGFR-Mutated Patients

**Figure 2:**
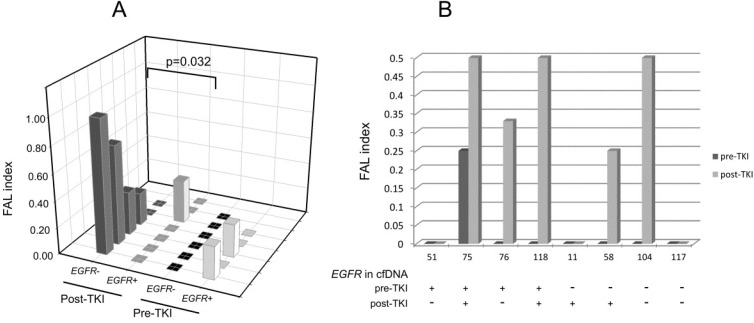
Loss of heterozygosity (LOH) frequency (fractional allelic loss (FAL) index). (**A**) *EGFR* mutation positive or negative cfDNA samples. *p*-Value was calculated using the Mann–Whitney test. (**B**) LOH frequency in pre- and post-tyrosine-kinase-inhibitor (TKI) matched samples.

Analysis of LOH in cfDNAs from the *EGFR*-mutated cohort showed that the median value of the fractional allelic loss (FAL) index—calculated by dividing the number of LOH-positive loci by the total number of informative loci—was 0 (Figure 2), and the FAL value did not change when we considered *EGFR*+ and *EGFR*− patients separately (Figure 2). Interestingly, by stratifying cfDNA samples in pre- and post-TKI treatment, we found that the *EGFR*− post-TKI subgroup had higher LOH frequency than all other subgroups (median FAL index 0.29 vs. 0). Indeed, as reported in Figure 2, among *EGFR*−pre-TKI cfDNA samples, no alterations were observed (median FAL index = 0); similarly, the median FAL index of *EGFR*+ pre- and post-TKI samples was 0, although two samples in the pre-TKI and one in the post-TKI had LOH at *TP53* locus. On the contrary, in *EGFR*− post-TKI samples, we observed LOH in four out of six cfDNAs (median FAL index = 0.29); interestingly, one sample showed microsatellite instability (MSI) in D3S1234 (*FHIT*). Despite the limited number of cfDNA samples analyzed, the frequency of LOH alterations was significantly higher in *EGFR*− post-TKI samples compared with *EGFR*+ post-TKI samples (*p* = 0.032, Mann–Whitney). Notably, no association between LOH and/or MSI frequencies and smoking habits of patients or concentration of cfDNA in plasma was found. Finally, intrigued by the apparent increase of LOH frequency in post-TKI samples, we analyzed LOH in eight matched plasma samples collected either pre-TKI or at progression following TKI administration. As shown in Figure 2B, LOHs were detected in five out of eight post-TKI cfDNA samples, and the FAL index of patient no. 75, who was already positive to LOH, further increased. Interestingly, LOH detection was uncoupled from detection of *EGFR* mutations in plasma by qPCR (Figure 2B). These results, albeit preliminary, suggest that TKI therapy might select cancer populations bearing increased levels of genetic instability.

### 2.3. Analysis of LOH in KRAS-Mutated Patients

**Figure 3:**
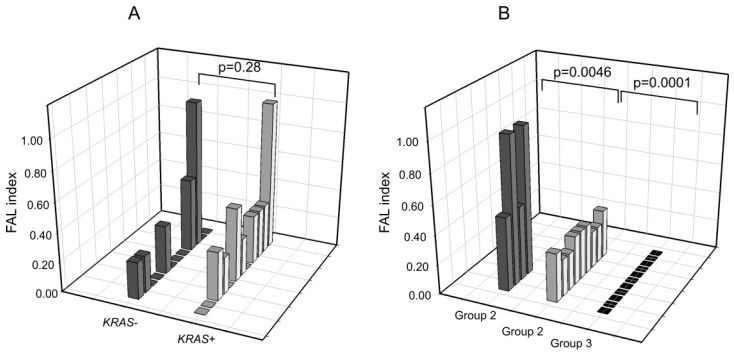
LOH distribution in *KRAS* mutation positive or negative cfDNA samples. (**A**) FAL index comparison between *KRAS*+ and *KRAS*−. (**B**) Stratifications of samples based on their LOH frequency. *p*-Values were calculated using the Mann–Whitney test.

With regard to the *KRAS*-mutated cohort, no significant differences (*p* = 0.28) were observed between *KRAS*+ and *KRAS*− cfDNA samples, although *KRAS*+ samples exhibited a slightly higher alteration frequency (Figure 3A). Based on the number of alterations, samples could be arbitrarily stratified into three subgroups. Group 1 disclosed a high LOH pattern (median FAL index: 0.75), group 2 disclosed an intermediate LOH frequency (median FAL index: 0.33), and group 3 lacked detectable alterations at the analyzed markers (median FAL index: 0) (Figure 3B). Among these three groups, differences in the FAL index were significant, with *p*-values ranging from 0.0046 (group 1 vs. group 2) to 0.0001 (group 2 vs. group 3).

### 2.4. LOH Frequency Comparison between EGFR+ and KRAS+ cfDNA Samples

**Figure 4:**
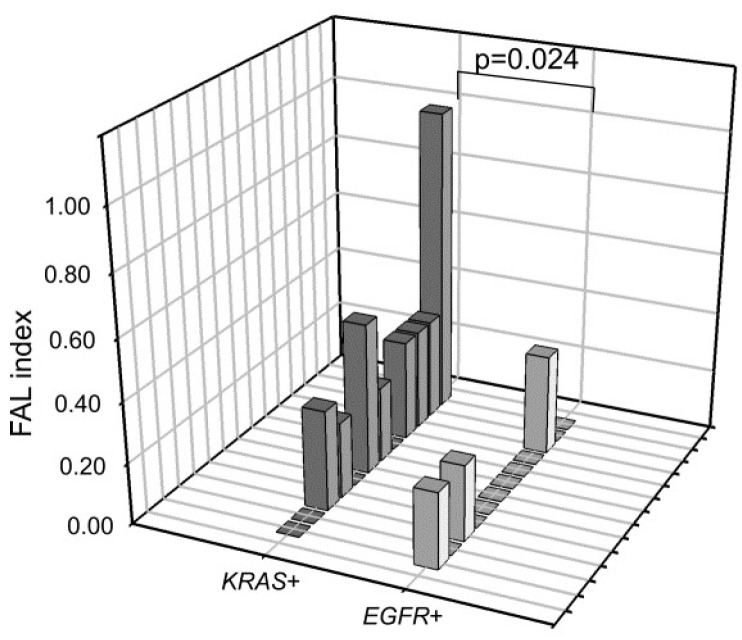
LOH frequencies in cfDNA samples positive for *EGFR* and *KRAS* mutations. *p*-Value was calculated using the Mann–Whitney test.

We next compared the LOH FAL index in *EGFR*+ and *KRAS*+ cfDNAs and found that *KRAS*+ samples had significantly more alterations than *EGFR*+ samples (*p* = 0.024) (Figure 4). On the contrary, no statistical significance was observed between *EGFR*− and *KRAS*− samples (*p* = 0.82), probably due to the relatively high LOH frequency of *EGFR*− compared with *EGFR*+ samples (Figure 2).

### 2.5. LOH Comparison between Plasma and Tumor Specimens

**Figure 5:**
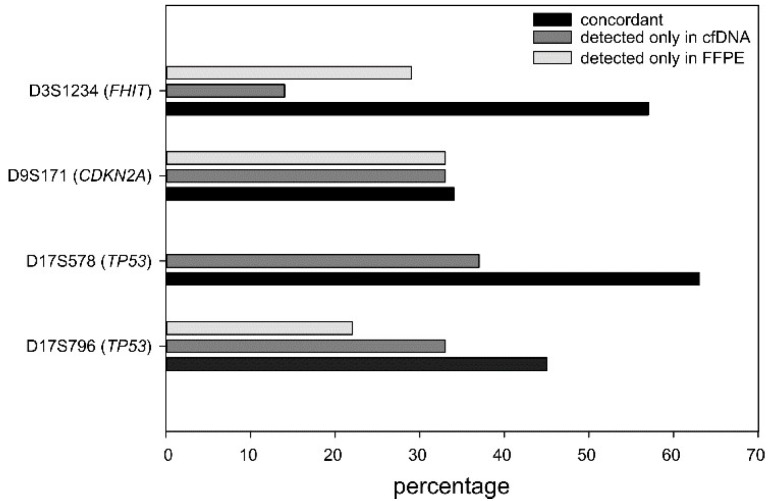
Concordance between alterations detected in cfDNA and formalin-fixed paraffin-embedded (FFPE)-DNA at the single marker level.

To investigate the concordance between the alterations detected in cfDNAs with those present in tumor, we analyzed 10 time-matched formalin-fixed paraffin-embedded (FFPE)-DNA samples. Considering all MS markers together, we found a median concordance of 50%, with two patients showing a 100% concordance and eight patients a concordance ranging between 25% and 75%. When the MS markers were considered individually, we observed that concordance of D9S171 (*CDKN2A* locus), D17S796 (*TP53* locus), and D3S1234 (*FHIT*) ranged from 34% to 57%. On the contrary, D17S578 (*TP53* locus) exhibited a concordance of >60%, and the discrepant samples all resulted in altered cfDNA (Figure 5). Despite the limited number of samples analyzed, these results might reflect the potential of cfDNA analysis to investigate tumor heterogeneity.

## 3. Discussion

The translational research on liquid biopsy in *EGFR*-mutated lung cancer is still debating how a negative result should be interpreted. The prevailing view is that lack of *EGFR* mutation detection in the cfDNA of patients with advanced *EGFR*-mutated NSCLC might reflect low amounts of circulating tumor DNA, which can depend on various factors, including tumor burden, location of primary tumor and its metastasis, as well as an intrinsic nonshedder feature of the tumor. Moreover, some noninformative results may clearly depend on the still limited sensitivity of routinely used molecular assays. These considerations do not exclude the possibility that in some patients, additional genetic alterations, which are not routinely tested and therefore invisible, might accumulate in cfDNA. These alterations could stem from *EGFR*-mutated tumor cells but also from tumor clones lacking *EGFR* mutation. Furthermore, recent studies have uncovered that in patients with multiple pulmonary nodules, which represent up to 10% of all lung cancers, a sizeable quota are independent tumors, which disclose a completely different genetic makeup [19,20].

Our pilot study interrogated the presence of LOH at certain lung-cancer-associated MS markers in cfDNA from NSCLC patients bearing either *EGFR*- or *KRAS*-mutated tumors. Results are of manifold interest in this context. The most valuable result is that the frequency of LOH alterations was significantly higher in *EGFR*− post-TKI samples compared with both *EGFR*+ pre- and post-TKI samples (Figure 2A). This result is important because it suggests that at tumor progression following TKI administration, there is a positive selection of other cancer populations presumably lacking *EGFR* mutations that accumulate LOH events. This accumulation is likely driven by TKI treatment. Evaluation of LOH alterations in eight matched pre- and post-TKI cfDNA samples further reinforced this working hypothesis (Figure 2B). These results are in line with a recent work by Jin et al., who reported increased chromosomal instability in patients who underwent *EGFR*-specific TKI treatment [21].

Even though a control group carrying a different tumor-specific genetic alteration and not receiving targeted therapy was included, the total number of samples analyzed remains small, and these results need to be confirmed in an independent study.

The second interesting result is that, while in the case of *EGFR*-mutated samples, LOH alterations were substantially more common in cfDNA *EGFR*− post-TKI samples, in the case of *KRAS*-mutated samples, no clear association was found between *KRAS* status in cfDNA and LOH events (Figure 3A). The latter were overall more common in *KRAS*-mutated samples than in *EGFR*-mutated samples, likely due to the different smoking habits of these two cohorts. Finally, it was interesting to observe that among *KRAS*-mutated samples, three distinct groups with different FAL frequencies can easily be distinguished (Figure 3B), and it will be interesting to further investigate whether the three groups are associated with a different clinical courses and whether the group with the highest FAL (group 1) is also characterized by a high tumor mutational burden. Indeed, since it is well known that LOH unmasks the presence of mutations in the remaining allele [18], samples with a high FAL index could potentially carry a higher number of genetic alterations. In this scenario, it will be interesting to explore the possible correlation between high FAL values in cfDNA and response to immune checkpoint inhibitors.

In the case of *KRAS* mutant samples, all cfDNA samples were collected prior to systemic therapy, and for this reason, it was not possible to investigate whether or not nontargeted therapy affects the frequency of LOH alterations.

Evaluating the concordance data between alterations in cfDNA and FFPE-DNA, we can conclude that among the nonconcordant cases, the majority showed more alterations in cfDNA. This finding could be artefactual but could also indicate the higher representativeness of the ctDNA of the tumor heterogeneity compared with the single FFPE specimen.

## 4. Materials and Methods

### 4.1. Patients and Samples

Circulating cfDNA samples of 48 patients with advanced NSCLC were included in this work; as a control, 10 time-matched tumor specimens were also analyzed. Twenty-four cfDNAs were selected from the EMULATING study. EMULATING was a monoinstitutional observational study, the primary end-point of which was to evaluate the concordance of *EGFR* mutation status between tumor tissue and cfDNA in terms of detection of both activating and resistance mutations. In this study, the overall sensitivity of the *EGFR*-specific PCR test was 56%. As a control population, 24 cfDNAs were selected among those positive for *KRAS* mutation in the MAGIC-1 study. MAGIC-1 was a monoinstitutional study which prospectively enrolled *EGFR*, *ALK1*, or *ROS1* wild-type advanced NSCLC patients. The primary end-point was to evaluate the sensitivity of detection in cfDNA of mutations previously identified in tumor tissue biopsy; in the case of *KRAS*, the sensitivity of the ddPCR assay was 50%. Both studies received authorization from the Ethics Committee of the Istituto Oncologico Veneto (IOV-IRCCS) (approval nos. 2015/35 and 26/03/2015 for EMULATING and nos. 2016/82 and 12/12/2016 for MAGIC-1). All patients gave their informed consent for the study.

### 4.2. Sample Collection and DNA Extraction

Twenty milliliters of blood were collected in EDTA tubes (BD Diagnostics, Buccinasco, Italy) and processed within 2 h or in Helix cfDNA stabilization tubes (Diatech Pharmacogenetics Srl, Jesi, Italy) and processed within 24–72 h. Plasma was separated by centrifugation at 2000× *g* for 10 min at 4 °C; to remove any cellular debris, plasma was centrifuged a second time at 16,000× *g* for 10 min at 4 °C and then stored at −80 °C until cfDNA extraction. Before plasma separation, 1 mL of whole blood was removed and stored at −20 °C for constitutive genomic DNA extraction.

### 4.3. DNA Extraction

cfDNA was extracted from 3 mL of plasma using the QIAmp^®^ circulating nucleic acid (QIAgen, Milano, Italy) or from 1 mL of plasma using the Maxwell^®^ RSC ccfDNA Plasma Kit (Promega, Madison, WI, USA), according to manufacturer’s instructions. The quality of the cfDNA samples was assessed with an Agilent 2100 Bioanalyzer System using the Agilent High-Sensitivity DNA Kit (Agilent Technologies, Santa Clara, CA, USA). Reference germline DNA was extracted from peripheral blood leukocytes with the QIAamp DNA Minikit (Qiagen, Milan, Italy) or with the automated extractor MagNA Pure Compact Instrument using the MagNA Pure Compact Nucleic Acid Isolation Kit I (Roche, Monza, Italy). DNA quantity and quality were measured with a NanoDrop 1000 spectrophotometer (Thermofisher, Monza, Italy). Tumor DNA was extracted from FFPE samples using the QIAamp DNA Micro Kit (Qiagen, Milan, Italy) according to manufacturer’s instructions. A pathologist identified tumor areas and, when possible, macrodissection was done. Depending on the quality of the biopsy specimen, the tumor fraction ranged from 10% to 75%.

### 4.4. Genetic Analyses

Tumor tissue genotyping and assessment of *EGFR* and *KRAS* status was performed using the Sequenom MassARRAy^®^ (Sequenom MA, USA) Myriapod Lung Status Kit (Diatech Pharmacogenetics SRL, Jesi, Italy) or NGS using a custom TruSeqCustom Amplicon panel (Illumina) within the framework of routine molecular diagnostic protocols. Detection of common *EGFR* mutations in exons 19–21 in plasma samples was assessed by scorpion-ARMS real-time PCR, using EASY *EGFR* real time (Diatech), and by ddPCR. Detection of *KRAS* mutations in codons 12, 13, and 61 in plasma samples was performed by ddPCR. ddPCR was carried out on the QX200 ddPCR system (Bio-Rad Laboratories, Milano, Italy), and the specific probes were purchased from Bio-Rad.

### 4.5. LOH and MSI Analysis

LOH and MSI were investigated in cfDNAs and time-matched FFPE-DNA samples using a panel of four MSs: D3S1234 in chr. 3p14.2 internal to *FHIT*, D9S171 in chr. 9p21.3 at 2.5 Mb downstream of *CDKN2A/2B*, D17S796 in chr. 17p13.2, and D17S578 in chr. 17p13.1 at 1.3 and 0.7 Mb upstream of *TP53*, respectively. Selection of these MSs was based on (i) their chromosomal position nearby or within suppressor genes frequently lost in tumors, (ii) their high frequency of heterozygosity in the Caucasian population (i.e., high informativeness), and (iii) the small size of the amplification product, which is crucial to successfully amplify low-quality DNA such as cfDNA. Locations of MSs and primer sequences were obtained from the UCSC Genome Browser (Human December 2013 GRCh38/hg38 Assembly). PCR primers and conditions have been previously described [22]. Forward primers were 5′-end labeled with FAM or HEX fluorescent dies (Sigma-Aldrich, Milan, Italy) and PCR products were analyzed using the 3730xl DNA analyzer (Life Technologies, Monza, Italy). Informativeness (i.e., heterozygosity) of a given MS was assessed for each patient in the germline DNA, and LOH analysis was performed only for patients heterozygous for the locus. LOH was defined as a reduction of the fluorescent signal in one allele of the sample compared to the germline DNA, while the presence of new alleles assessed MSI positivity. In LOH analysis studies, a cut-off of 30% reduction is acceptable to state LOH positivity [23,24]. To be more stringent, we set the cut-off at ≥35% for both cfDNA and FFPE-DNA samples. All cfDNAs were analyzed in duplicate and data reproducibility was evaluated by performing reanalysis of a few randomly chosen samples. To estimate the global alteration status of each patient, we calculated the FAL index by dividing the number of LOH-positive loci by the total number of informative loci. Only patients that were heterozygous (informative) for at least two markers were considered for the FAL index calculation.

### 4.6. Data Analysis and Statistics

Statistical analyses were performed using MedCalc software, version 12.2.1 (MedCalc Software, Ostend, Belgium). Statistical tests were two-sided. The Mann–Whitney *U* test was performed for the FAL index comparison to estimate difference at a global level between groups. Kruskal–Wallis one-way analysis of variance on ranks was used for comparison between subgroups in *KRAS*-mutated samples.

## 5. Conclusions

In conclusion, our pilot study indicates the feasibility of detecting LOH events in common lung-cancer-associated MS markers in cfDNA from advanced NSCLC patients. Our findings suggest an easy-to-perform and economically sustainable assay to integrate mutation-specific high-sensitivity assays in the case of *EGFR*-mutated patients, as well as a candidate molecular assay to interrogate genomic alterations and their association with response to immunotherapy in *KRAS*-mutated patients.

## Figures and Tables

**Table 1:** Clinical features of NSCLC patients.

**(A) Clinical Features of EGFR-Mutated Patients**		
**Age at Diagnosis (years)**	**Median (range)**	**70 (43–87)**
Gender	Male	13 (54%)
	Female	11 (46%)
Smoking	No	13 (54.2%)
	Current	0 (0%)
	Former	11 (45.8%)
PS	0	6 (25%)
	1	18 (75%)
	2	0 (0%)
Stage at Diagnosis	I–II	0 (0%)
	III–IV	24 (100%)
cfDNA *EGFR* test	Diagnosis	11 (50%)
	Progression Disease	13 (50%)
**Total**		**24**
**(B) Clinical Features of *KRAS*-Mutated Patients**		
**Age at Diagnosis (years)**	**Median (range)**	**69.5 (48–85)**
Gender	Male	11 (46%)
	Female	13 (54%)
Smoking	No	4 (16.7%)
	Current	9 (37.5%)
	Former	11 (45.8%)
PS	0	8 (33%)
	1	15 (63%)
	2	1 (4 %)
Stage at Diagnosis	I–II	0 (0%)
	III–IV	24 (100%)
cfDNA *KRAS* test	Diagnosis	22 (92%)
	Progression Disease	2 (8%)
**Total**		**24**

Abbreviations: PS—performance status; cfDNA—cell-free DNA; *EGFR*—epidermal growth factor receptor.
